# Metabolic profiling identifies trehalose as an abundant and diurnally fluctuating metabolite in the microalga *Ostreococcus tauri*

**DOI:** 10.1007/s11306-017-1203-1

**Published:** 2017-04-17

**Authors:** Matthias Hirth, Silvia Liverani, Sebastian Mahlow, François-Yves Bouget, Georg Pohnert, Severin Sasso

**Affiliations:** 1 Institute of General Botany and Plant Physiology, Friedrich Schiller University, Jena, Germany; 2 Department of Mathematics, Brunel University London, Uxbridge, UK; 3 Sorbonne Universités, UPMC Univ Paris 06 & Centre National pour la Recherche Scientifique CNRS, UMR 7621, Laboratoire d’Océanographie Microbienne, Observatoire Océanologique, Banyuls-sur-Mer, France; 4 Institute for Inorganic and Analytical Chemistry, Friedrich Schiller University, Jena, Germany; 5 Max Planck Institute for Chemical Ecology, Jena, Germany

**Keywords:** Microalgae, Picoeukaryotes, Carbon metabolism, Untargeted metabolomics, pantothenate.

## Abstract

**Introduction:**

The picoeukaryotic alga *Ostreococcus tauri* (Chlorophyta) belongs to the widespread group of marine prasinophytes. Despite its ecological importance, little is known about the metabolism of this alga.

**Objectives:**

In this work, changes in the metabolome were quantified when *O. tauri* was grown under alternating cycles of 12 h light and 12 h darkness.

**Methods:**

Algal metabolism was analyzed by gas chromatography-mass spectrometry. Using fluorescence-activated cell sorting, the bacteria associated with *O. tauri* were depleted to below 0.1% of total cells at the time of metabolic profiling.

**Results:**

Of 111 metabolites quantified over light–dark cycles, 20 (18%) showed clear diurnal variations. The strongest fluctuations were found for trehalose. With an intracellular concentration of 1.6 mM in the dark, this disaccharide was six times more abundant at night than during the day. This fluctuation pattern of trehalose may be a consequence of starch degradation or of the synchronized cell cycle. On the other hand, maltose (and also sucrose) was below the detection limit (~10 μM). Accumulation of glycine in the light is in agreement with the presence of a classical glycolate pathway of photorespiration. We also provide evidence for the presence of fatty acid methyl and ethyl esters in *O. tauri*.

**Conclusions:**

This study shows how the metabolism of *O. tauri* adapts to day and night and gives new insights into the configuration of the carbon metabolism. In addition, several less common metabolites were identified.

**Electronic supplementary material:**

The online version of this article (doi:10.1007/s11306-017-1203-1) contains supplementary material, which is available to authorized users.

## Introduction

Photosynthesis has profoundly changed life on Earth and is vitally important for all aerobic organisms today. In the world oceans, phytoplankton is responsible for the fixation of ~45–50 gigatons of carbon per year (Falkowski et al. 1998). Aquatic photosynthesis is at the origin of the fossil fuels we exploit today, and at the same time contributes to the sequestration of carbon from the atmosphere (Falkowski et al. 1998). Understanding the life of photosynthetic organisms is pivotal for a description of microbial communities, food webs and biogeochemical cycles (Worden et al. 2015).

Marine picoeukaryotes, which have a diameter of 2 µm and less, make significant contributions to primary production in coastal and oligotrophic waters (Li 1994; Worden et al. 2004). Owing to their large surface area/volume ratios, picoeukaryotes have a competitive advantage over larger cells at low external concentrations of nutrients (Falkowski et al. 1998). Among the picophytoplankton, the order Mamiellales contains several cosmopolitan genera including *Ostreococcus, Micromonas*, and *Bathycoccus* (Vaulot et al. 2008). These Mamiellophyceae (Chlorophyta) represent a phylogenetically deep-branching class within the green lineage (Leliaert et al. 2012). While *Ostreococcus tauri* persists at densities of ~2 × 10^5^ cells ml^−1^ in the Mediterranean Thau Lagoon (Chrétiennot-Dinet et al. 1995), *Ostreococcus* spp. have also been found in other places such as the Atlantic and Pacific Oceans, where blooms have been observed (Derelle et al. 2006; O’Kelly et al. 2003; Collado-Fabbri et al. 2011). *Ostreococcus* strains that were sampled from different ocean depths showed adaptations to different light intensities (Rodríguez et al. 2005).

With a tiny cell size of only ~1 µm, the non-flagellated *O. tauri* cells feature a simple architecture with a single chloroplast and mitochondrion (Courties et al. 1994; Chrétiennot-Dinet et al. 1995). As a minimal version of a photosynthetic eukaryote, *O. tauri* has recently developed into a promising model organism of fundamental biology, and a variety of processes have been investigated including evolution (Blanc-Mathieu et al. 2013), viral infection (Derelle et al. 2008), photosynthesis (Cardol et al. 2008), light sensing (Heijde et al. 2010; Djouani-Tahri et al. 2011a), circadian rhythm (Corellou et al. 2009; O’Neill et al. 2011), cell division (Moulager et al. 2010), nutrient uptake (Botebol et al. 2015; Lelandais et al. 2016), fatty acid biosynthesis (Vaezi et al. 2013), and the regulation of starch metabolism (Sorokina et al. 2011). So far, *O. tauri* has only been observed in the form of haploid cells (Corellou et al. 2009; Grimsley et al. 2010). Its sequenced genome has a size of merely 12.6 Mb, accompanied by a decreased gene redundancy compared to the genomes of land plants (Derelle et al. 2006). In version 2, the genome sequence of *O. tauri* was improved by additional Illumina DNA and RNA sequencing, reassembly and reannotation (Blanc-Mathieu et al. 2014). Genetic transformation of *O. tauri* is established, and a high frequency of homologous recombination in the nucleus allows for tailored genetic modifications (Corellou et al. 2009; Lozano et al. 2014).

Algae regularly interact with bacteria, and these interactions can influence the productivity of microbial communities (Hom et al. 2015; Ramanan et al. 2016). A region called the phycosphere, which surrounds an algal cell, contains a gradient of algal metabolites that can affect the growth of other microorganisms (Amin et al. 2012). Possible explanations for the often observed difficulty to obtain axenic algal cultures are metabolic interconnections or physical contact between the interacting organisms (Abby et al. 2014; Hom et al. 2015). Despite sustained efforts to remove the bacteria contained in *O. tauri* laboratory cultures (Abby et al. 2014), there are currently no axenic *O. tauri* cultures available. A recent study highlighted that *O. tauri* can obtain a thiazole-containing precursor of vitamin B_1_ from *Marinobacter* sp. (Paerl et al. 2017). Even though *O. tauri* is auxotrophic for B_1_ (Paerl et al. 2017), this auxotrophy cannot explain the hurdles to eliminate the bacteria because growth media are commonly supplemented with this vitamin. Nevertheless, the difficulties to obtain axenic cultures of *O. tauri* might be a consequence of similar yet unknown trophic interactions with associated bacteria. The bacterial community associated with the originally sequenced *O. tauri* strain OTTH0595, used in this work, is dominated by *Marinobacter* spp. (γ-Proteobacteria) (Lupette et al. 2016; Paerl et al. 2017Lupette et al. 2016, 2017). Various other bacteria have been identified in this culture, including members of the Flavobacteriia and α-, β- and γ-Proteobacteria (Abby et al. 2014).

As a tiny member of the marine plankton, *O. tauri* is frequently exposed to changing environmental conditions, including variable illumination due to day and night or variable solar irradiation, changes in temperature, salinity, nutrient availability, or grazing pressure. Adaptation to variable abiotic and biotic conditions is accomplished with the help of ~120 transcription factors (Weirauch et al. 2014). So far, no experimental studies have systematically explored *O. tauri*’s metabolism, and its integration into the cell’s regulatory networks is incompletely understood. As a first step to describe the metabolism of this minimalist photosynthetic eukaryote, we applied untargeted quantitative metabolic profiling to *O. tauri* grown under light–dark cycles. Our strategy, which is based on gas chromatography-mass spectrometry (GC-MS), provides new insights particularly into the primary metabolism of this alga.

## Materials and methods

### Solvents and standard compounds

The following solvents were used for extraction and derivatization of metabolites: chloroform (Product no. 83626.290, VWR, Darmstadt, Germany), ethanol (1.11727.1000, VWR), methanol (34860, Sigma–Aldrich, Munich, Germany), and pyridine (270407, Sigma–Aldrich). Standard compounds are listed in Supplementary Table 1.

### Cultivation of *Ostreococcus tauri*

*Ostreococcus tauri* OTTH0595, which is sometimes called OTH95 (Courties et al. 1994; Derelle et al. 2006), was used for all experiments. RCC745 refers to the same strain, while RCC4221 is derived from it (Abby et al. 2014). *O. tauri* was cultivated in artificial sea water (ASWO medium), which is a modified Keller medium (Djouani-Tahri et al. 2011b). In addition to Keller trace metal solution (Keller et al. 1987), ASWO medium contains 420 mM NaCl, 10 mM KCl, 20 mM MgCl_2_, 10 mM CaCl_2_, 25 mM MgSO_4_, 2.5 mM NaHCO_3_, 0.88 mM NaNO_3_, 5.0 × 10^−5^ M NH_4_Cl, 1.0 × 10^−5^ M *β*-glycerophosphate, 1.0 × 10^−8^ M H_2_SeO_3_, 1 ml of 1 M Tris–HCl (pH 7.2) per liter of medium, 3.7 × 10^−10^ M cyanocobalamin, 2.0 × 10^−9^ M biotin, and 3.0 × 10^−7^ M thiamine. For metabolic profiling, 42 Erlenmeyer flasks (500 ml) containing 200 ml of ASWO medium each were inoculated to a cell density of 0.27 × 10^6^ cells ml^−1^. Cultures were incubated under alternating light–dark cycles, consisting of 12 h of blue light followed by 12 h of darkness, at a temperature of 19.0 ± 0.3 °C. Blue light of a final photosynthetic photon flux density of 10.6 ± 0.9 µmol photons m^−2^ s^−1^ was generated by passing white light (Philips Master TL-D 36 W 865 lamps) through a blue filter (Lee Filter Roll 183 Moonlight Blue, Thomann, Burgebrach, Germany). Cultures were agitated manually once a day.

### Antibiotic treatment

In an attempt to remove bacteria from *O. tauri* cultures, ASWO medium containing 310 μg ml^−1^ penicillin G (P3032, Sigma–Aldrich), 1.9 μg ml^−1^ chloramphenicol (C1919, Sigma–Aldrich), 1.4 μg ml^−1^ polymyxin B (P4932, Sigma–Aldrich), and 2.3 μg ml^−1^ neomycin (N1142, Sigma–Aldrich) was inoculated with *O. tauri*. After cultivation for 1 day, 220 μg ml^−1^ cefotaxime (C7039, Sigma–Aldrich) and 500 μg ml^−1^ carbenicillin (C3416, Sigma–Aldrich) were added. After six further days, the grown culture was used to inoculate fresh ASWO medium without antibiotics, and after several rounds of subcultivation, the bacterial content was quantified by analytical flow cytometry. A single antibiotic treatment decreased the number of bacteria in *O. tauri* cultures for several months, but was unable to remove them completely.

### Flow cytometry

To quantify algal and bacterial cells in *O. tauri* mixed cultures, aliquots of cells were fixed for 30 min with 0.25% (v/v) glutaraldehyde (3778.1, Carl Roth, Karlsruhe, Germany) at room temperature and stored at −20 °C until analysis. After thawing, SYBR Green I (S7563, Thermo Fisher Scientific, Darmstadt, Germany) was added to a 1× final concentration together with a defined number of counting beads (C36950, Thermo Fisher Scientific) for absolute quantification. A FACSCalibur flow cytometer (BD Biosciences) was used to analyze algal and bacterial cells using channels FL1 (530/30) and FL3 (670LP), and the counting beads using channels FL2 (585/42) and FL4 (661/16). Thresholds were adjusted to reduce background events in the FL1 and FL3 channels. For data analysis, the software Cell Quest (BD Biosciences, version 3.3) was used. The suitability of flow cytometry to quantify the density of *O. tauri* cells was verified by the parallel counting of cells using an improved Neubauer counting chamber (T729.1, Carl Roth, Karlsruhe, Germany) under a light microscope with 400-fold magnification (five technical replicates per biological replicate).

Fluorescence-activated cell sorting (FACS) of live cells was performed with a FACSAria Fusion instrument (BD Biosciences) equipped with an 85-μm diameter nozzle and controlled by the program BD FACSDiva version 8.0.1. Phosphate-buffered saline (A1286301, Thermo Fisher Scientific) was used as the sheath fluid. For sorting, an *O. tauri* culture with a density of ~10^7^ cells ml^−1^ and a bacterial content of 11.2% was used. Cells were selected based on a suitable population in a scatter plot of the channels FSC (488/10) and B695 (695/40). 6 × 10^4^ sorted cells were spun down (5 min, 4000 x*g*) and used to inoculate a fresh culture with an initial density of 5.5 × 10^3^ cells ml^−1^ in ASWO medium. After several rounds of subcultivation, the bacterial content was reassessed by analytical flow cytometry.

### Acquisition of metadata (inorganic nutrients, pH)

Fifty millilitres of culture were sterile-filtered, frozen and sent to the GEOMAR Helmholtz Centre for Ocean Research in Kiel (Germany). After thawing, samples were tenfold diluted with NaCl solution (salinity of 36) and measured in triplicates. Nitrate, nitrite and ammonium were determined photometrically (Hansen and Koroleff 1999). As a standard, a solution containing 80 µM nitrate, 2.0 µM nitrite and 5.0 µM ammonium was used. pH was determined with digital pH meter pH525 equipped with electrode SenTix 21 (WTW, Weilheim, Germany).

### Metabolite sampling and derivatization

To sample *O. tauri* metabolites, we largely followed a previously established protocol (Vidoudez and Pohnert 2012): Cells were collected by filtration of 150 ml of culture through a pre-washed Whatman GF/F filter (Z242519, Sigma–Aldrich) under reduced pressure (800 mbar). The cells were lysed by addition of 3.5 ml of extraction mix optimized for microalgae (methanol:ethanol:chloroform 2:6:2, Vidoudez and Pohnert 2012) and incubation for 5 min at room temperature. For time points during darkness, sampling was performed under green light. As a negative control, 150 ml of ASWO medium were filtered, the filter washed with extraction mix, and the resulting sample processed in the same way as the main samples. Extracts were stored at −80 °C until further use.

For derivatization, insoluble material was removed by centrifugation (16,000×*g*, 4 °C, 5 min), and 20 nmol of ribitol (internal standard) were added to a volume equivalent of 4.8 × 10^9^ cells (as determined by flow cytometry). After samples were dried at 50 mbar in a desiccator, the residues were dissolved in 100 µl pyridine containing 2 mg of methoxyamine hydrochloride (226904, Sigma Aldrich) and vortexed for 1 min. Samples were incubated at 60 °C for 1 h, then at room temperature for 17 h. For the second derivatization step, 40 μl of retention index mix (decane, pentadecane, nonadecane, octacosane, dotriacontane all 1 mM, and hexatriacontane 0.5 mM in hexane, all >99%, Sigma–Aldrich) were added to 1 ml of *N*-methyl-*N*-(trimethylsilyl)trifluoroacetamide (MSTFA, 701270, Macherey–Nagel, Düren, Germany), and 50 μl of this mixture were added to 50 μl of sample and incubated at 40 °C for 1 h. Afterwards, the samples were centrifuged (3000×*g*, 4 °C, 10 min), and the supernatant analyzed by GC-MS.

### GC-MS

Samples were analyzed in random order on a TRACE Ultra gas chromatograph (Thermo Scientific) equipped with an AS3000 II auto sampler and a DB-5MS Agilent column (30 m x 0.250 mm x 0.25 µm) with a 10 m Duraguard pre-column. The GC was connected to an ISQ Single Quadrupole mass spectrometer (Thermo Scientific). For each batch of 20 samples, a new deactivated glass liner (5 mm inner diameter, 8 mm outer diameter, 105 mm length; Thermo Scientific) with glass wool was used. Liners were shipped to CS-Chromatographie Service (Langerwehe, Germany) for cleaning and deactivation (GW INNO-Sil Plus). 1 μl of sample was injected at 300 °C and analyzed in split mode 10. The oven temperature was kept at 60 °C for 1 min, then increased by 15 °C min^−1^ to 310 °C and kept at 310 °C for 10 min. The flow rate of the helium 5.0 carrier gas was set to 1 ml min^−1^. Ionization and fragmentation was performed by an electron impact source at 70 eV and 280 °C. Five MS scans per second were recorded with a resolution of 866 at *m*/*z* 502.20 (FWHM = 0.53).

### Data processing, quantification of metabolites, and data analysis

GC-MS data files (*.raw) were converted to *.cdf files using the file converter of Xcalibur 2.2.44 (Thermo Scientific). The remaining data processing was done as described by Vidoudez and Pohnert 2012, with the following modifications/details: AMDIS version 2.70 (http://www.amdis.net) was used, and component width was set to 12; version 2.08 of program MET-IDEA (Broeckling et al. 2006) was used with the parameter MS type: quadrupole. The resulting datasets were imported into Excel 2010 (Office 2010, Microsoft, Redmont, USA). Using extracted ion chromatograms (EICs) of model ions provided by AMDIS, peak areas were normalized by the peak area of the internal standard ribitol (*m*/*z* 103.1 at 11.66 min). Peaks were further considered only if they were present in all samples, and if their intensities were at least fivefold over background. For this purpose, the average peak area was compared to the fivefold average peak area of the negative controls. The resulting data were double-checked to eliminate errors in peak integration and redundant model ions that originate from the same compound. Bayesian Fourier clustering of temporal metabolite patterns was performed as described previously for transcript patterns (Monnier et al. 2010).

### Identification of metabolites

For each quantified metabolite, mass spectra at the peak maximum of the EIC were background-corrected using a region adjacent to the peak in the same EIC. The resulting spectrum was compared with mass spectra deposited in the NIST/EPA/NIH Mass Spectral Library version 2011 using the software MS Search (version 2.0d from 2005; http://chemdata.nist.gov/mass-spc/ms-search). The identification of selected metabolites was verified with the help of retention indices and mass spectra of authentic standards (Supplementary Table 1) analyzed under identical conditions. Platform-independent retention indices were determined with the help of the retention times of the unknown metabolite and those of two alkanes in the retention index mix (cf. Sect. 2.5) that elute before and after the unknown metabolite. To calculate non-isothermal retention indices, the following equation derived from Lee et al. 1979 was used:

$$I=100\;\left[ {n+\left( {N - n} \right)\frac{{t_{r}\left( {unknown} \right) - t_{r}\left( n \right)}}{{t_{r}\left( N \right) - t_{r}\left( n \right)}}} \right]$$

where *I* is the retention index, *n* is the number of carbon atoms of the alkane eluting earlier, *N* is the number of carbon atoms of the alkane eluting later, and *t*_r_ is the retention time. The unknown metabolite was considered identical with the authentic standard if their retention indices differed by no more than five units, and the mass-spectral match value was at least 700.

For the identification of trehalose in *O. tauri*, metabolites were extracted as described in Sect. 2.5, but using 200 ml of a 9-day old culture (~10^10^ cells). The dried extract was dissolved in 2 ml 20 mM potassium phosphate buffer, pH 6.0. For comparison, 100 nmol of trehalose, maltose or glucose (supplemented with internal standard) were used (Supplementary Table 1). Samples were incubated with 0.08 units of maltase (G9259-100UN, Sigma–Aldrich) or trehalase (E-TREH, Megazyme, Bray, Ireland) for 1 h at 37 °C. Reactions were quenched by addition of 2 ml of chloroform followed by 10 s of vortexing. The aqueous phase was dried under nitrogen gas, and samples were derivatized and analyzed as described in Sects. 2.5 and 2.6.

## Results

### Flow-cytometric analysis of *O. tauri* cultures

**Fig. 1:**
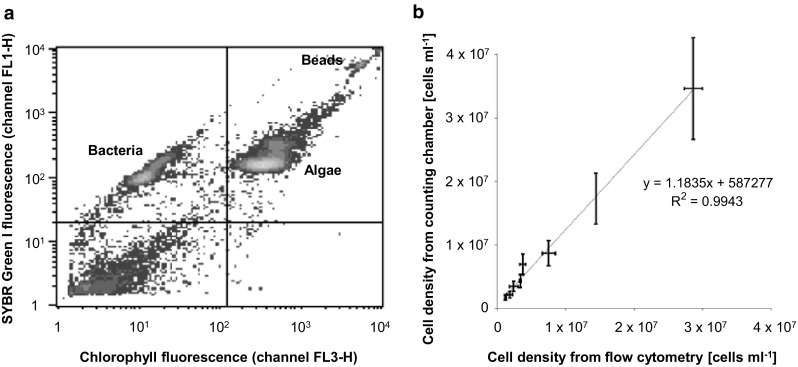
Quantification of cell density and bacterial content of *O. tauri* cultures by flow cytometry. **a** Example of a flow cytogram of an *O. tauri* culture (density plot). For illustration, a sample with a significant portion of bacteria (20%) is shown. **b** Cell densities obtained by flow cytometry compared to cell densities obtained by hemocytometer counting of different dilutions of 16-day old *O. tauri* cultures. In both cases, total cells were counted. For the flow-cytometric measurements, samples with the indicated cell densities were prepared with ASWO medium, and two technical replicates were counted. For hemocytometry, three independent biological samples with a cell density of ~7 × 10^6^ cells ml^−1^ were counted (using five technical replicates per biological replicate), and the cell densities of the remaining samples were calculated. Mean ± standard deviation is shown

As a first step towards the metabolic profiling of the marine microalga *O. tauri*, we used flow cytometry to simultaneously quantify the cell densities of algae and bacteria present in the same culture. For this purpose, cells were fixed with glutaraldehyde and stained with the DNA-binding dye SYBR Green I (see Sect. 2 for details). Samples were then analyzed by flow cytometry using one channel that detects SYBR Green I fluorescence, and one channel that detects chlorophyll fluorescence (Fig. 1a). In the resulting flow cytogram, *O. tauri* cells are represented by events that show high levels of both chlorophyll and bound SYBR Green I, while non-photosynthetic cells (mostly bacteria) only show a high amount of SYBR Green I (Fig. 1a).

Algal densities obtained by flow cytometry correlated well with values obtained by hemocytometry (Fig. 1b). Quantification by flow cytometry was applicable to cell densities between 1.2 × 10^6^ and 2.9 × 10^7^ cells ml^−1^ (Fig. 1b), covering a dynamic range of at least 25-fold.

This experiment confirms previous studies (Chrétiennot-Dinet et al. 1995; Moulager et al. 2010) that flow cytometry is a suitable method to quantify the cell density of *O. tauri* cultures quickly and reliably, and it shows how algal and bacterial cells can be distinguished.

There are currently no axenic *O. tauri* cultures available. We thus attempted to establish such a culture using a cocktail of six different antibiotics (see Sect. 2), but failed to remove the bacteria completely. As an alternative strategy to obtain an axenic culture, fluorescence-activated cell sorting (FACS) was applied to an *O. tauri* culture not previously treated with antibiotics. While the complete removal of bacteria was impossible, FACS successfully depleted the bacteria from ~10% to below 1% (values indicate the number of bacteria relative to the total number of cells). This bacterial content appeared sufficiently low to exclude substantial effects of the bacteria on the metabolic profile. As a consequence of FACS sorting, the bacterial content remained low throughout the metabolic profiling experiment (see below).

### Metabolic profiling: experimental design and collection of metadata

**Fig. 2:**
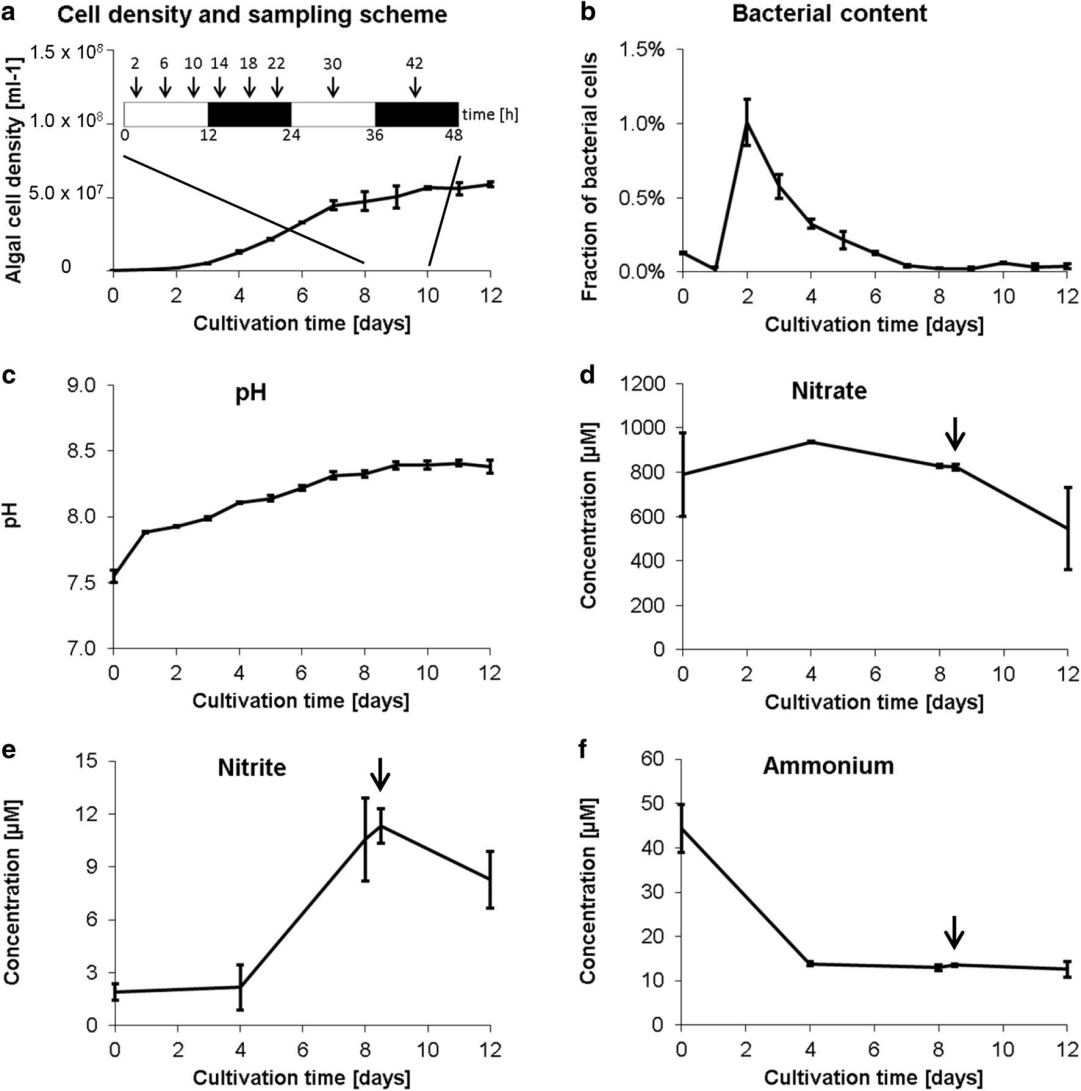
Experimental design for metabolic profiling of *O. tauri* under 12 h light:12 h dark conditions and collection of metadata. **a** Time points for the sampling of intracellular metabolites (*arrows*) and growth curve. To quantify algal cell density, samples were taken in the middle of the day (LD6). Note that data collected for an additional time point (42 h) were discarded due to normalization problems. **b**–**f** Other metadata collected over the course of the experiment including bacterial content of *O. tauri* cultures (**b**), pH (**c**), and the concentrations of nitrate (**d**), nitrite (**e**), and ammonium (**f**). The samples were taken in the middle of the day (LD6) except the time point marked by an arrow, which was taken in the middle of the night (LD18). Mean ± standard deviation of biological triplicates is shown (except for t = 1 day and 2 days in (**a**–**c**), and t = 0 in (**d**–**f**), where duplicates where measured)

*Ostreococcus* isolates have been found in waters from a variety of depths and trophic states. The *O. tauri* isolate OTTH0595 used in this study originates from surface waters of a shallow Mediterranean lagoon and is able to acclimate to a wide range of light intensities (Rodríguez et al. 2005). To monitor how the metabolism of *O. tauri* responds to changing light conditions, algae were grown under 12 h light–12 h dark cycles, and metabolites were sampled every 4 h during late exponential/early stationary phase (Fig. 2a). In parallel, a variety of metadata were sampled to characterize the basic growth conditions during this experiment. *O. tauri* cultures reached early stationary phase after 7 days, and from then onwards, the cell density stayed relatively constant (~5 to 6 × 10^7^ cells ml^−1^) until the end of the experiment (Fig. 2a). During the cultivation period of 12 days, the bacterial content briefly peaked at ~1.0% 2 days after inoculation before dropping back below 0.1%. During the time of metabolite sampling (between days 8 and 10), the bacterial values were between 0.02 and 0.06% (Fig. 2b). pH slowly but continuously increased from 7.9 at day 1 to 8.4 at day 12 (Fig. 2c). It appears that the algae used ammonium as their major nitrogen source during the first ~4 days before they switched to nitrate, as judged from the decrease in nitrate that started on day 4 (Fig. 2d), a concomitant increase in nitrite (Fig. 2e), and the decrease in ammonium that leveled off after day 4 (Fig. 2f).

### Profiling and identification of *O. tauri* metabolites

For metabolic profiling, extracts of *O. tauri* cells were derivatized with methoxyamine hydrochloride and *N*-methyl-*N*-(trimethylsilyl)trifluoroacetamide (MSTFA) and analyzed by GC-MS (see Sect. 2). Deconvolution of individual peaks in total ion current (TIC) chromatograms using program AMDIS (http://www.amdis.net) yielded a list of model ions for the detected compounds. For these model ions, EICs were generated, and the resulting peaks were quantified with the help of program MET-IDEA (Broeckling et al. 2006). Peak areas were normalized using ribitol added as an internal standard to each extract sample. After manual curation, data processing yielded 111 quantifiable peaks with intensities at least fivefold over background. While the majority of these 111 peaks correspond to distinct algal metabolites, a few peaks may correspond to alternative derivatization products derived from the same algal metabolite. For example, metabolites #6 and #11 (Table 1) are both derived from proline, carrying one and two trimethylsilyl modifications, respectively.

**Table 1:** Overview of intracellular metabolites identified in *O. tauri* and confirmed with the help of authentic standards

Metabolite number	Metabolite	Class	Retention index^a^	*m*/*z* of model ion	Match value^b^
3	alanine	amino acid	1102	116.2	836
6	proline^c^	amino acid	1188	129.0	821
7	3-chloro-1,2-propanediol	glycerol derivative	1192	116.0	728
11	proline^c^	amino acid	1307	142.1	920
12	glycine	amino acid	1314	174.2	766
14	glycerate	sugar acid	1332	189.0	761
16	pyrrole 2-carboxylate	heteroaromatic carboxylic acid	1358	240.0	836
20	*β*-alanine	amino acid	1429	248.0	828
22	pyroglutamate^d^	amino acid	1518	156.1	893
27	threonate	sugar acid	1547	292.2	924
31	proline^c^	amino acid	1582	142.2	847
41	methyl tetradecanoate	fatty acid ester	1733	143.2	907
55	phytol^e^	diterpene alcohol	1842	123.1	930
56	phytol^e^	diterpene alcohol	1864	123.2	923
57	phytol^e^	diterpene alcohol	1882	109.1	944
61	methyl palmitate	fatty acid ester	1929	143.1	920
64	ethyl palmitate	fatty acid ester	2008	101.1	760
71	methyl stearidonate	fatty acid ester	2115	105.0	924
72	methyl linolenate	fatty acid ester	2132	108.1	865
78	phytol^e^	diterpene alcohol	2202	143.1	882
80	linolenate (C18:3)	fatty acid	2253	107.2	902
93	methyl docosahexaenoate	fatty acid ester	2494	105.0	909
96	1-palmitoylglycerol	fatty acid ester	2603	371.3	805
98	trehalose	disaccharide	2736	361.1	909
99	squalene	triterpene	2816	121.2	898

Parent compounds of detected derivatives are listed. Metabolites were only considered if peaks were present in all samples, and if peak intensities were at least fivefold over background (see Sect. 2 for details). A full list of identified metabolites is given in Supplementary Table 1

^a^The retention index was calculated from average retention times of the compound of interest and the two alkanes that elute before and after it

^b^Match values are given for the comparison with an authentic standard analyzed on the same GC-MS instrument

^c^The authentic standard proline yielded a set of peaks that included the peaks observed for metabolites #6, #11 and #31

^d^Pyroglutamate is formed from glutamate during GC sample injection (Vidoudez and Pohnert 2012)

^e^The authentic standard phytol yielded a set of peaks that included the peaks observed for metabolites #55, #56, #57 and #78

To identify metabolites, the corresponding spectra were compared to spectra deposited in the NIST/EPA/NIH Mass Spectral Library (file NIST11_MSMS_2012.iso) using program MS Search (version 2.0d, http://chemdata.nist.gov/mass-spc/ms-search). 74 tentatively identified compounds with a match value higher than 600 are shown in Supplementary Table 2; of these, 31 compounds had a match value above 800, indicative of good confidence. To increase the reliability of these identifications, a set of authentic standards (Supplementary Table 1) was run on the same GC-MS system. Of totally 53 standards, 20 compounds were present in *O. tauri* cells (Table 1), whereas 33 compounds could not be detected in algal extracts.

The compounds identified with high confidence include a variety of amino acids, fatty acid esters and other primary metabolites (Table 1). In agreement with the notion that plants are capable of pantothenate (vitamin B_5_) biosynthesis (Smith et al. 2007), *β*-alanine (metabolite #20) was detected in *O. tauri*. This is further supported by the presence of genes for ketopantoate hydroxymethyltransferase (ostta02g02520) and pantothenate synthetase (ostta09g02630). The function of threonate (#27), which we also identified in *O. tauri*, is poorly understood, but this compound is possibly a product of ascorbate catabolism (DeBolt et al. 2007). Pyrrole 2-carboxylate (#16), also found in the algae, might be a chlorophyll catabolite, but it should be mentioned that chlorophyll degradation commonly stops at the stage of linear tetrapyrroles (Hörtensteiner and Kräutler 2011). While 3-chloro-1,2-propanediol (#7) was also detected, it is difficult to tell whether this compound really exists in *O. tauri*. Alternatively, it is possible that 3-chloro-1,2-propanediol was formed from the glycerol moiety of lipids during the derivatization procedure. We did not detect any sucrose in *O. tauri*.

### *O. tauri* metabolites showing diurnal fluctuations

**Fig. 3:**
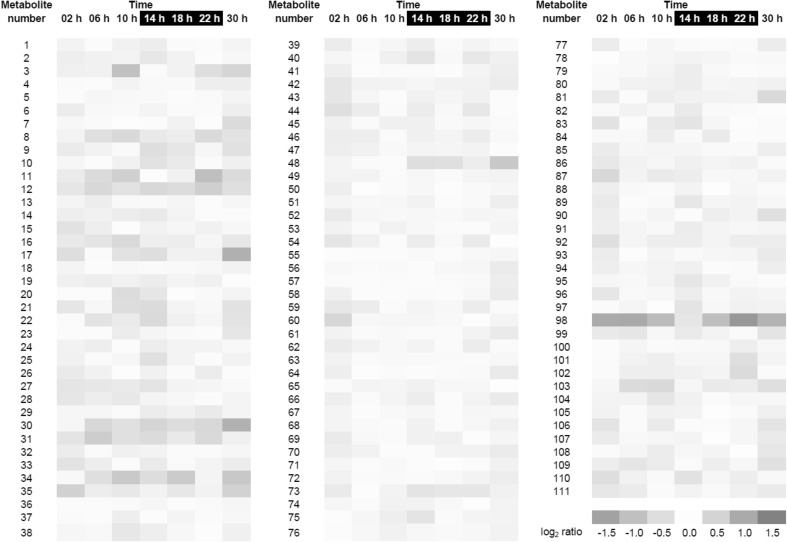
Time course of *O. tauri* metabolite concentrations over a light–dark cycle (heat map). For the metabolites of 111 quantifiable peaks, log_2_ values of mean abundances were calculated and centered on zero. *Blue* indicates increased metabolite abundance, red indicates decreased abundance. The underlying sampling scheme is depicted in Fig. 2a, and the absolute values used to prepare this figure are provided in Supplementary Table 3

Figure 3 graphically depicts the temporal changes of the metabolites that correspond to the 111 quantifiable peaks. To identify diurnally oscillating compounds, we analyzed how the concentrations of the different metabolites varied over a light–dark cycle. For this purpose, the temporal patterns of all quantifiable peaks were grouped into clusters using a Bayesian Fourier clustering method, which was previously used for analyzing RNA microarray data (Monnier et al. 2010). The metabolites grouped into five clusters (Supplementary Fig. 1). The abundances of the metabolites from cluster 1 show no or only small fluctuations. Cluster 2 contains metabolites that are more abundant during the day, while clusters 3, 4 and 5 contain metabolites that are more abundant during the night. In other words, 20 of 111 metabolites (18%) group into clusters with clear diurnal fluctuations.

**Fig. 4:**
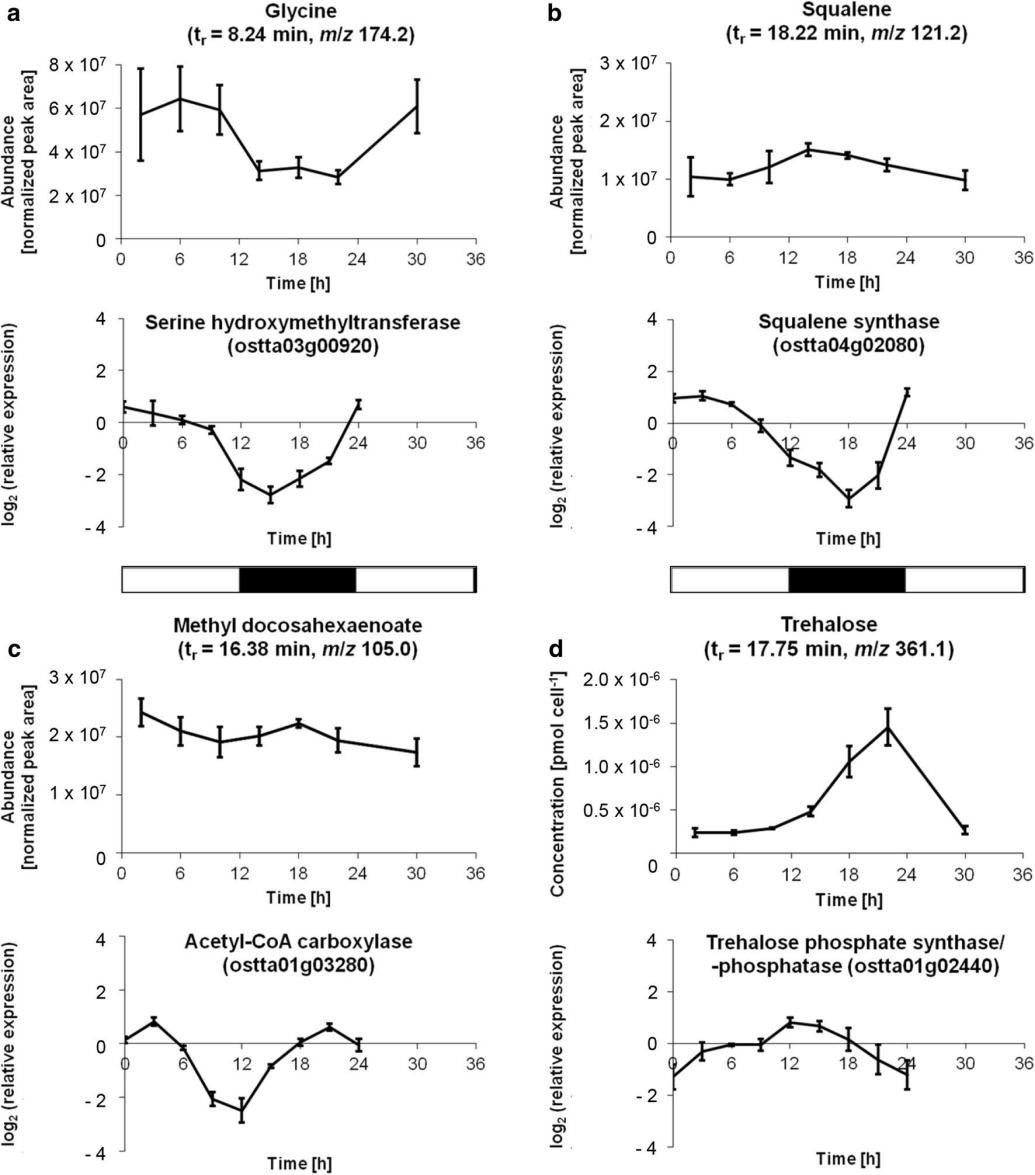
Selected intracellular metabolites that show diurnal fluctuations in *O. tauri*. **a** Glycine (#12). **b** Squalene (#99). **c** Methyl docosahexaenoate (#93). **d** Trehalose (#98). For the metabolites, which were sampled according to the scheme shown in Fig. 2a, mean ± standard deviation of four biological replicates is shown. Since a standard curve was measured for trehalose (Supplementary Fig. 2), the trehalose concentration is expressed in pmol cell^−1^. For comparison, transcript levels (taken from Monnier et al. 2010) are depicted for selected genes related to the respective metabolites. The identifiers of all genes mentioned in this work are listed in Supplementary Table 4. The horizontal bars in the middle of the figure denote the light and dark phases (*white* and *black* areas, respectively)

Examples of diurnally fluctuating metabolites include glycine, which is up to 2.1-fold more abundant in the light than in darkness (Fig. 4a). Light accumulation of glycine (#12), an intermediate of the photorespiratory pathway, was also reported for *Arabidopsis thaliana* (Gibon et al. 2006) or the macroalga *Ectocarpus siliculosus* (Gravot et al. 2010). For comparison, transcriptome data from Monnier et al. 2010 were included in Fig. 4 for selected genes related to the metabolites shown. Of three genes predicted to encode serine hydroxymethyltransferase in *O. tauri*, the transcript levels of one gene parallel the levels of glycine (Fig. 4a) and may thus encode the isoform involved in photorespiration. These data are in agreement with the presence of a classical glycolate pathway of photorespiration in *O. tauri*.

The abundance of squalene (#99), the universal precursor of triterpenes including phytosterols, peaks in the early dark phase (Fig. 4b). This pattern contrasts with the transcript profile of the single squalene synthase gene, which is highest in the early light phase (Fig. 4b). Therefore, even though squalene synthase serves as the first committed step in triterpene biosynthesis, this maximal shift indicates that the transcript levels of the squalene synthase gene in *O. tauri* is a poor indicator for the levels of its enzymatic products.

The levels of the methyl ester of docosahexaenoic acid (DHA, C22:6) (#93) reached a maximum at night (Fig. 4c). While the amplitude of this fluctuation is low, this pattern is similar to the daily patterns of other confirmed fatty acid esters (metabolite numbers 41, 61, 64, 71, 72 and 96; Fig. 3 and Supplementary Table 3). This profile roughly coincides with the transcript levels of the gene for the committed step of fatty acid biosynthesis (acetyl-CoA carboxylase), which features pronounced peaks at night and early morning (Fig. 4c). In contrast, many free fatty acids accumulate to some extent around dusk in *A. thaliana*, even though eicosenoate (C20:1), for example, peaks at night (Gibon et al. 2006).

Finally, we found the strongest fluctuations for trehalose (#98), which is up to sixfold more abundant in the night compared to the day (Fig. 4d). The identity of this non-reducing disaccharide was confirmed with the help of authentic trehalose (Table 1).

### Trehalose accumulates to a concentration of ~1.6 mM at night

**Fig. 5:**
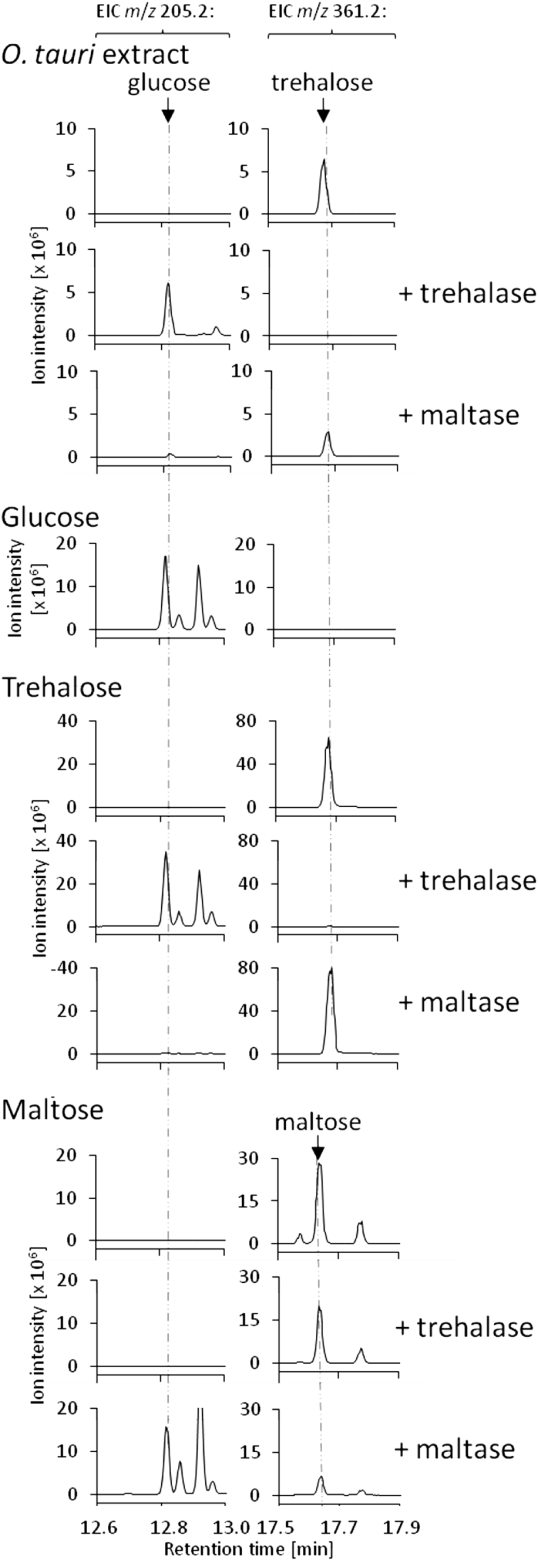
Verification of trehalose in *O. tauri*. Shown are peaks from extracted ion chromatograms (EICs) representative of trehalose, maltose, or glucose. To unambiguously identify the disaccharide from *O. tauri* detected by GC-MS, an extract was treated with either trehalase or maltase. Only trehalase, but not maltase, efficiently hydrolyzed the disaccharide into glucose, showing that *O. tauri* produces trehalose. For comparison, standards of trehalose and maltose (enzyme-treated or untreated) and glucose are shown

Since the discovery of the strong day/night fluctuations of trehalose (Fig. 4d) in *O. tauri* was somewhat surprising, we aimed at further corroborating the identity of this metabolite. For example, maltose also consists of two glucose monomers and had a retention time (~17.64 min) very similar to that of trehalose (~17.68 min). We thus treated an extract of *O. tauri* with trehalase, which resulted in the complete disappearance of the trehalose peak and the concomitant appearance of the cleavage product glucose (Fig. 5). In contrast, treatment with maltase only resulted in a minor amount of glucose (Fig. 5), further confirming that *O. tauri* indeed produces trehalose. As a control, authentic samples of trehalose and maltose were also treated with each of the two enzymes (Fig. 5).

To determine the absolute amounts of trehalose inside *O. tauri* cells, a standard curve was measured (Supplementary Fig. 2). Intracellular trehalose concentrations ranged from 0.23 × 10^−6^ pmol cell^−1^during the light phase to 1.45 × 10^−6^ pmol cell^−1^ in the dark phase (Fig. 4d). Using an average cell volume of 0.91 µm^3^, which was determined at the end of the dark phase (Henderson et al. 2007), the highest trehalose concentration is 1.60 mM. Since this calculation assumes a uniform intracellular distribution of trehalose, actual concentrations may even be higher if trehalose is accumulated in specific compartments. Taken together, we have found strong fluctuations of trehalose over light–dark cycles, which peak at night with millimolar concentrations.

## Discussion

As a vitally important source of energy, light dominates many aspects in the lives of photosynthetic organisms. The adaptation of plant metabolism to diurnal cycles has been studied by quantitative metabolic profiling in several plants, including *A. thaliana* (Gibon et al. 2006), rice (Sato et al. 2008) and *Nicotiana attenuata* (Kim et al. 2011), and the brown alga *E. siliculosus* (Gravot et al. 2010). Under a photoperiod of 12 h light and 12 h darkness, cells of the photosynthetic eukaryote *O. tauri* divide in a synchronized fashion at the end of the light phase or the beginning of the dark phase (Farinas et al. 2006). Using this light regime, we have quantified the levels of 111 metabolites in *O. tauri* using GC-MS. Since metabolic profiling was performed during late exponential/early stationary phase (Fig. 2a), a low rate of cell division and a relatively constant average cell volume are expected during our experiment. Supported by the use of standards, 25 metabolites were confirmed with high confidence (Table 1), and 49 additional metabolites were tentatively identified using database comparisons (Supplementary Table 2). A relatively high number of metabolites had low match values and remained unidentified (Supplementary Table 2). This may reflect the fact that the metabolism of *O. tauri* has been poorly characterized so far, and suggest the presence of a number of uncommon metabolites.

As in previous attempts of antibiotic treatment (Abby et al. 2014), we were unable to remove associated bacteria from *O. tauri* completely using an antibiotic cocktail or FACS. These observations might be explained by a physical association between algae and bacteria, but further work is required to investigate this possibility. The bacteria that remained in the culture after FACS sorting are expected to influence the metabolite profiles to some degree. We however conclude that removal of these apparently essential associated microbes would cause stress to *O. tauri* to a degree that its metabolome would essentially only reflect stress conditions and mask natural physiological responses. We therefore carried out our study with a substantially reduced load of associated bacteria and summarize the detected metabolites under the term *O. tauri* metabolome.The low content of bacteria at the point of metabolite sampling (below 0.1% of total cells) suggests only small contributions by the bacteria. It is evident that the presence of bacteria may affect the regulation of algal metabolism, and it will not be possible to quantify this effect until axenic cultures of *O. tauri* become available. In some cases, microbial communities even acquire the capacity for the biosynthesis of novel metabolites that cannot be produced by the individual microorganisms on their own (cooperative biosynthesis); so far however, only few examples of this phenomenon are known (Hom et al. 2015; Kusari et al. 2016).

Of the fatty acid esters identified in *O. tauri*, several were confirmed with the help of standards (Table 1). For example, we found both methyl palmitate (#61) and ethyl palmitate (#64, eluting ~0.45 min after methyl palmitate), whereas free palmitate could not be detected. Even when pure palmitic acid was dissolved in extraction mix (methanol:ethanol:chloroform 2:6:2) before derivatization, only the monosilylated derivative was detected by GC-MS (eluting ~0.31 min after ethyl palmitate), but no alkyl esters. These findings suggest that *O. tauri* produces a range of fatty acid esters, even though we cannot rule out with complete certainty that the detected fatty acid esters were formed during the derivatization by esterification of free fatty acids or transesterification of lipid-bound fatty acids. Evidence for the biosynthesis of fatty acid esters also exists in *Chlamydomonas reinhardtii* (Herrera-Valencia et al. 2012), another chlorophyte distantly related to *O. tauri*.

With a sixfold peak-to-peak amplitude, trehalose showed the strongest fluctuation of all metabolites quantified in *O. tauri* grown under light–dark cycles (Fig. 4d). Similarly, *A. thaliana* produces a diurnally oscillating disaccharide that was not identified (Kim et al. 2011), but could be trehalose. Trehalose is made by all organisms except vertebrates (Paul et al. 2008). Plants generally use trehalose phosphate synthase (TPS) to convert glucose 6-phosphate and UDP-glucose into trehalose 6-phosphate, which is then converted into trehalose by trehalose phosphatase (TPP) (Lunn et al. 2014). The genome of *O. tauri* encodes two bifunctional TPS-TPP versions (identifiers ostta01g02440 and ostta12g02400) and one monofunctional TPP (ostta14g00250) (Blanc-Mathieu et al. 2014). While the transcript of the monofunctional TPP is most abundant during the day, both bifunctional TPS-TPP genes are upregulated in the first half of the night (Fig. 4d and Supplementary Fig. 3), which is largely in agreement with a pathway that uses two monosaccharide building blocks to make trehalose. Our dataset does not allow for any statements on the levels of sugar phosphates such as trehalose 6-phosphate, possibly because their derivatives do not reach the mass spectrometer.

In plants and algae, trehalose and its precursor trehalose 6-phosphate have been implicated in a variety of functions ranging from carbon storage to the regulation of carbon metabolism, the protection from osmotic stress and other forms of stress, or signaling in plant–microbe interactions (Paul et al. 2008; Lunn et al. 2014; Michel et al. 2010). In vascular plants, the function of trehalose as a stress protectant has been replaced by sucrose (Lunn et al. 2014). In *Saccharomyces cerevisiae*, trehalose is a key factor that enables survival to long-term desiccation (Tapia and Koshland 2014). Since *O. tauri* can cope with a wide range of salinities ranging from 5 to 65‰ (F.-Y. Bouget, unpublished results), it is possible, for example, that trehalose enhances the survival of this marine alga under hyperosmotic stress. Accumulation of trehalose in *O. tauri* during the dark phase might be triggered by starch degradation. As in other plants and algae, starch is built up by *O. tauri* in the light until it reaches a maximum at dusk, and is then used as a source of energy and carbon in the night (Sorokina et al. 2011). In vascular plants, high levels of trehalose 6-phosphate increase the rate of starch biosynthesis via redox activation of ADP-glucose pyrophosphorylase (Kolbe et al. 2005). However, the observation that ADP-glucose pyrophosphorylase from *O. tauri* is not redox-regulated (Kuhn et al. 2009) argues against such a function of trehalose 6-phosphate in this alga. With an average intracellular concentration of 1.6 mM, trehalose reaches its highest concentration at the end of the night (Fig. 4d). This apparent delay compared to the starch peak may suggest that trehalose accumulation is a consequence of starch degradation. Based on a prediction by the program PredAlgo (Tardif et al. 2012), the trehalose-forming enzymes mentioned above are localized in either chloroplast (ostta01g02440) or cytosol (ostta12g02400 and ostta14g00250), which is consistent with such a scenario. A connection between trehalose accumulation and starch degradation is further supported by the apparent absence of trehalose from *E. siliculosus* (Gravot et al. 2010), which does not produce starch (Michel et al. 2010).

In contrast to the major products of starch degradation, the reducing sugars maltose and glucose (Streb and Zeeman 2012), trehalose is a non-reducing sugar and thus less reactive. In our analysis, maltose was not detected in any of the samples (Fig. 5 and data not shown). Based on an estimated detection limit of ~0.2 pmol, the intracellular concentration of maltose is below ~10 µM. It is plausible that *O. tauri* produces trehalose to prevent excessive amounts of reactive starch degradation products, with trehalose functioning as a temporary buffer of carbon and energy during starch degradation. Alternatively, the diurnal fluctuation of trehalose may be a consequence of the synchronized cell cycle. In *S. cerevisiae*, maximal intracellular trehalose concentrations were detected at the G1/S transition (Ewald et al. 2016). When trehalose peaks at the end of the dark phase (Fig. 4d), *O. tauri* is in the middle of the G1 phase (Farinas et al. 2006). Further investigations of the function of trehalose in *O. tauri* will be greatly supported by the generation and characterization of trehalose biosynthesis mutants.

## Electronic supplementary material

Below is the link to the electronic supplementary material.

Supplementary material 1 (DOCX 237 KB)

Supplementary material 2 (DOCX 69 KB)

Supplementary material 4 (DOCX 84 KB)

Supplementary material 4 (DOCX 17 KB)

Supplementary material 5 (DOCX 33 KB)

Supplementary material 6 (XLSX 33 KB)

Supplementary material 7 (DOCX 11 KB)
