# Supplementary material for: Metabolic profiling identifies trehalose as an abundant and diurnally fluctuating metabolite in the microalga Ostreococcus tauri
Source: Metabolomics. 2017 Apr 17;13(6):68. doi: 10.1007/s11306-017-1203-1 (PMC5392535; doi:10.1007/s11306-017-1203-1)
Supplement: Supplementary file 1 — Supplementary material 1 (DOCX 237 KB) [file 11306_2017_1203_MOESM1_ESM.docx]

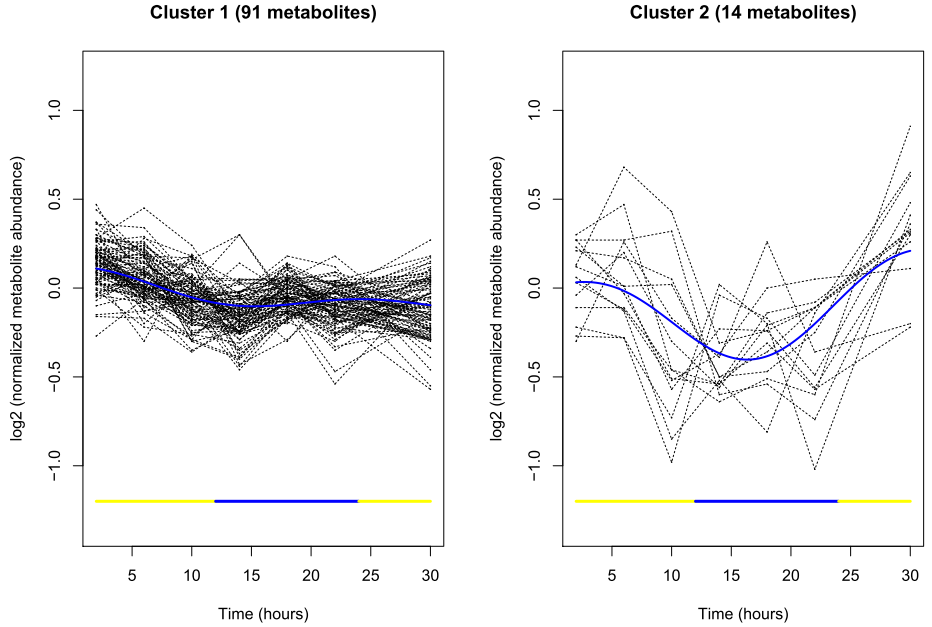


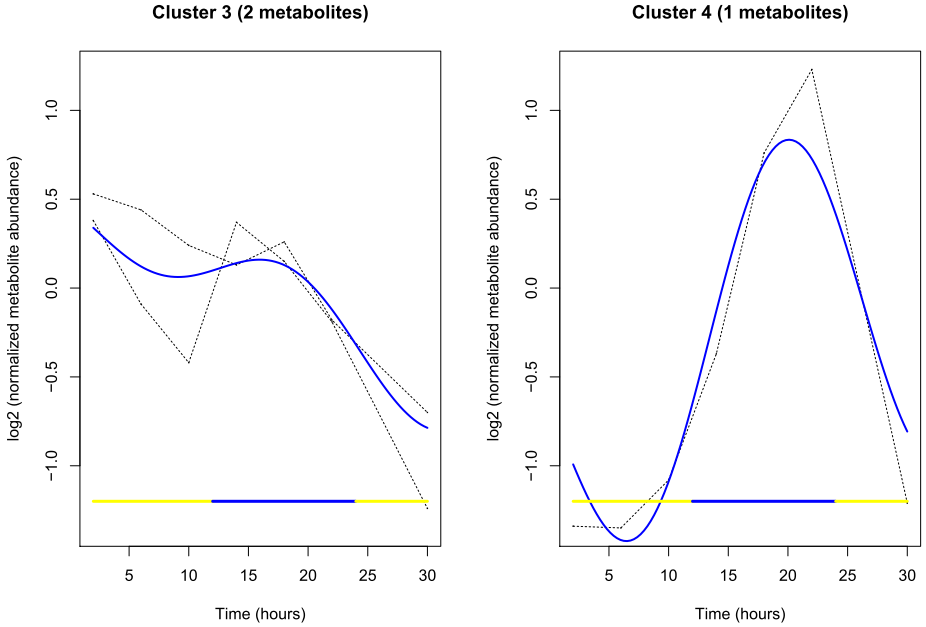


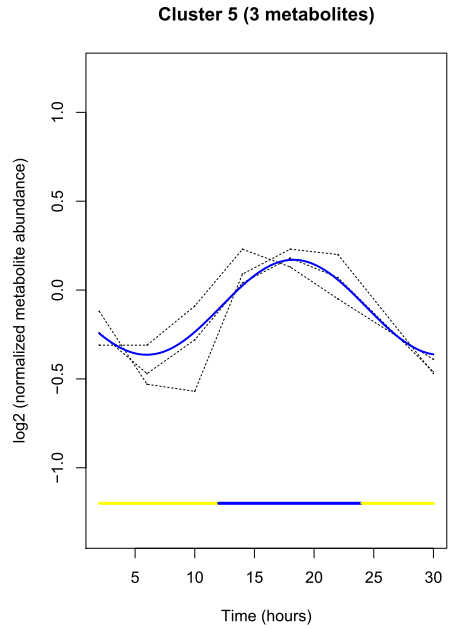


**Supplementary Fig. 1. Cluster analysis of metabolite time courses over a light-dark cycle.** The 111 quantifiable metabolites were grouped by Bayesian Fourier clustering. Cluster 1 contains 91 metabolites (no. 1, 2, 4-7, 10, 13-15, 18, 19, 23-29, 32, 33, 36-47, 49-72, 74-97, 100-102, 104-108, 110 and 111), cluster 2 contains 14 metabolites (no. 3, 8, 9, 11, 12, 16, 20, 21, 22, 30, 31, 34, 48 and 73), cluster 3 contains two metabolites (no. 17 and 35), cluster 4 contains one metabolite (no. 98), and cluster 5 contains three metabolites (no. 99, 103 and 109). The yellow vertical lines indicate the light phase, while the blue vertical lines indicate the dark phase.
