# Supplementary material for: Metabolic profiling identifies trehalose as an abundant and diurnally fluctuating metabolite in the microalga Ostreococcus tauri
Source: Metabolomics. 2017 Apr 17;13(6):68. doi: 10.1007/s11306-017-1203-1 (PMC5392535; doi:10.1007/s11306-017-1203-1)
Supplement: Supplementary file 2 — Supplementary material 2 (DOCX 69 KB) [file 11306_2017_1203_MOESM2_ESM.docx]

**Supplementary Fig. 2. Standard curve for the absolute quantification of trehalose.** Six different concentrations of a trehalose standard were mixed with a constant amount of the internal standard ribitol and analyzed by GC-MS. Trehalose concentrations are plotted against the peak area of trehalose (EIC of *m*/*z* 361.1) normalized by the peak area of ribitol (EIC of *m*/*z* 103.1). Data of duplicate samples are shown.
