# Supplementary material for: Metabolic profiling identifies trehalose as an abundant and diurnally fluctuating metabolite in the microalga Ostreococcus tauri
Source: Metabolomics. 2017 Apr 17;13(6):68. doi: 10.1007/s11306-017-1203-1 (PMC5392535; doi:10.1007/s11306-017-1203-1)
Supplement: Supplementary file 3 — Supplementary material 4 (DOCX 84 KB) [file 11306_2017_1203_MOESM3_ESM.docx]

**Supplementary Fig. 3. Diurnal transcript levels of additional genes for a bifunctional trehalose phosphate synthase-trehalose phosphatase (ostta12g02400) and a monofunctional trehalose phosphatase (ostta14g00250).** Transcript level data are from [Monnier et al. 2010](#_ENREF_42). See also Fig. 4d.
