# Supplementary material for: Metabolic profiling identifies trehalose as an abundant and diurnally fluctuating metabolite in the microalga Ostreococcus tauri
Source: Metabolomics. 2017 Apr 17;13(6):68. doi: 10.1007/s11306-017-1203-1 (PMC5392535; doi:10.1007/s11306-017-1203-1)
Supplement: Supplementary file 4 — Supplementary material 4 (DOCX 17 KB) [file 11306_2017_1203_MOESM4_ESM.docx]

**Supplementary Table 1. List of authentic standards used for the identification of metabolites**

| Standard | Supplier | Product number |
| --- | --- | --- |
|  |  |  |
| *β*-alanine | Sigma-Aldrich, Munich (Germany) | 05159-100G |
| alanine | Merck Millipore, Darmstadt (Germany) | 1010070025 |
| 2-amino-4,6-dihydroxypyrimidine | Sigma-Aldrich | A50401-25G |
| arachidonic acid | Sigma-Aldrich | A3611-100MG |
| *β*-carotene | Sigma-Aldrich | 22040-5G-F |
| 3-chloro-1,2-propanediol | Sigma-Aldrich | 107271-25ML |
| 5-*α*-cholestane | Sigma-Aldrich | C8003-100MG |
| 7-dehydrocholesterol | Sigma-Aldrich | 30800-5G-F |
| 2-deoxyribonic acid lithium salt | Sigma-Aldrich | 14699-10MG |
| 2-deoxy-D-ribose | Sigma-Aldrich | 121649-1G |
| *cis*-4,7,10,13,16,19-docosahexaenoic acid | Sigma-Aldrich | D2534-25MG |
| *cis*-4,7,10,13,16,19-docosahexaenoic acid methyl ester | Sigma-Aldrich | D2659-10MG |
| *cis*-5,8,11,14,17-eicosapentaenoic acid | Sigma-Aldrich | 44864-100MG |
| ergosta-5,7,9(11),22-tetraen-3β-ol | Sigma-Aldrich | E2634-5MG |
| ergosterol hydrate | Alfa Aesar, Karlsruhe (Germany) | B23840 |
| L-ergothioneine | Tetrahedron, Paris (France) | THD-201 |
| ethyl palmitate | Sigma-Aldrich | P9009-5G |
| 4-*O*-β-galactopyranosyl-D-mannopyranose | Sigma-Aldrich | G0886-25MG |
| geranylgeraniol | Sigma-Aldrich | G3278-100MG |
| glucose | Carl Roth, Karlsruhe (Germany) | X997.3 |
| glyceraldehyde | Sigma-Aldrich | 49800-1G |
| glyceric acid sodium salt | Sigma-Aldrich | 61786-10MG |
| glycerol | Carl Roth | 7530.2 |
| glycine | Alfa Aesar | A13816 |
| lactose monohydrate | Sigma-Aldrich | 61339-25G |
| linoleic acid | Alfa Aesar | L07949 |
| linolenic acid | Sigma-Aldrich | L2376-500MG |
| lyxose | Alfa Aesar | A17007 |
| maltose monohydrate | Sigma-Aldrich | 63418-25G |
| methyl α-glucopyranoside | Alfa Aesar | A12484 |
| methyl linolenate | Sigma-Aldrich | 62210-10ML-F |
| methyl myristate | Sigma-Aldrich | M3378-1G |
| methyl palmitate | Sigma-Aldrich | P5177-1G |
| 1-methyl-1-cyclohexanecarboxylic acid | Sigma-Aldrich | 142824-10G |
| *N*-methyldodecanamide | TCI Deutschland GmbH, Eschborn (Germany) | M2452 |
| nerol | MP Biomedicals Germany GmbH, Eschwege (Germany) | 0215581880 - 100 mL |
| palmitic acid | Merck Millipore | 8005080100 |
| 1-palmitoylglycerol (α-palmitin) | Sigma-Aldrich | M1640-100MG |
| 2-palmitoylglycerol | Santa Cruz Biotechnology, Inc., Heidelberg (Germany) | sc-203465 |
| pentadecanal | TCI Deutschland GmbH | P1869 |
| phytol (mixture of isomers) | Sigma-Aldrich | 139912-10G |
| proline | Sigma-Aldrich | P0380-100G |
| pyroglutamic acid | Sigma-Aldrich | P5960-25G |
| pyrrole-2-carboxylic acid | Sigma-Aldrich | P73609-1G |
| ribose | Alfa Aesar | A17894 |
| squalene | Sigma-Aldrich | S3626-10ML |
| stearidonic acid methyl ester | VWR International GmbH, Darmstadt (Germany) | CAYM10005000-1 |
| sucrose | Sigma-Aldrich | 84097-250G |
| threonic acid hemicalcium salt | Sigma-Aldrich | 380644-5G |
| trehalose dihydrate | Sigma-Aldrich | T9531-5G |
| turanose | Sigma-Aldrich | T2754-1G |
| valine | Sigma-Aldrich | V0500-100G |
| xylose | VEB Berlin Chemie, Berlin (Germany) |  |
