# Supplementary material for: Metabolic profiling identifies trehalose as an abundant and diurnally fluctuating metabolite in the microalga Ostreococcus tauri
Source: Metabolomics. 2017 Apr 17;13(6):68. doi: 10.1007/s11306-017-1203-1 (PMC5392535; doi:10.1007/s11306-017-1203-1)
Supplement: Supplementary file 5 — Supplementary material 5 (DOCX 33 KB) [file 11306_2017_1203_MOESM5_ESM.docx]

**Supplementary Table 2. List of intracellular metabolites detected in *O. tauri*.** Parent compounds of detected derivatives are listed. Metabolites were only considered if peaks were present in all samples, and if peak intensities were at least fivefold over background (see Materials and methods).

Footnotes:

^1)^ For metabolites not confirmed with an authentic standard, the following abbreviations are used: ?, match value ≥ 800; ??, 600 ≤ match value < 800; ???, match value < 600; ^*)^, standard was measured, but did not agree with prediction from database comparison

^2)^ The retention index was calculated from average retention times of the compound of interest and the two alkanes that elute before and after it.

^3)^ Match values are given for the comparison with an authentic standard analyzed on the same GC-MS instrument, or for the comparison with the spectrum in the NIST/EPA/NIH Mass Spectral Library if no data on authentic standards were available.

^4)^ The authentic standard proline yielded a set of peaks that included the peaks observed for metabolites #6, #11 and #31.

^5)^ Most likely a contamination from sample processing

^6)^ Pyroglutamate is formed from glutamate during GC sample injection ([Vidoudez and Pohnert 2012](#_ENREF_58))

^7)^ The authentic standard phytol yielded a set of peaks that included the peaks observed for metabolites #55, #56, #57 and #78.

| Metabolite number | Metabolite^1)^ | Class | Retention time (min) | Retention index^2)^ | *m*/*z* of model ion | Match value^3)^ | Verified with authentic standard? |
| --- | --- | --- | --- | --- | --- | --- | --- |
|  |  |  |  |  |  |  |  |
| 1 | 2-chloroethyl ethyl sulfide ?? | sulfide | 4.93 | n.d. | 124.0 | 616 | no |
| 2 | ethylene glycol ?? | alcohol | 5.04 | n.d. | 125.2 | 656 | no |
| 3 | alanine | amino acid | 6.22 | 1102 | 116.2 | 836 | yes |
| 4 | dodecamethylpentasiloxane ?? ^5)^ | siloxane | 6.81 | 1164 | 174.1 | 734 | no |
| 5 | ??? |  | 6.90 | 1173 | 152.0 |  | no |
| 6 | proline^4)^ | amino acid | 7.04 | 1188 | 129.0 | 821 | yes |
| 7 | 3-chloro-1,2-propanediol | glycerol derivative | 7.08 | 1192 | 116.0 | 728 | yes |
| 8 | 1,1,1,5,5,5-hexamethyl-3,3-bis(trimethylsiloxy)trisiloxane ?? ^5)^ | siloxane | 7.20 | 1205 | 170.1 | 611 | no |
| 9 | serine ?? | amino acid | 7.78 | 1265 | 146.2 | 676 | no |
| 10 | ??? |  | 8.08 | 1297 | 165.0 |  | no |
| 11 | proline^4)^ | amino acid | 8.18 | 1307 | 142.1 | 920 | yes |
| 12 | glycine | amino acid | 8.24 | 1314 | 174.2 | 766 | yes |
| 13 | lavandulol ?? | monoterpene alcohol | 8.38 | 1328 | 157.1 | 734 | no |
| 14 | glycerate | sugar acid | 8.42 | 1332 | 189.0 | 761 | yes |
| 15 | ??? |  | 8.46 | 1337 | 196.1 |  | no |
| 16 | pyrrole-2-carboxylate | heteroaromatic carboxylic acid | 8.66 | 1358 | 240.0 | 836 | yes |
| 17 | nonanoate (C9:0) ?? | fatty acid | 8.74 | 1366 | 189.1 | 675 | no |
| 18 | threonine ?? | amino acid | 8.90 | 1383 | 157.1 | 771 | no |
| 19 | pyridine ?? | heteroaromatic compound | 9.09 | 1402 | 243.1 | 682 | no |
| 20 | *β*-alanine | amino acid | 9.34 | 1429 | 248.0 | 828 | yes |
| 21 | ??? |  | 9.87 | 1485 | 228.0 |  | no |
| 22 | pyroglutamate^6)^ | amino acid | 10.15 | 1518 | 156.1 | 893 | yes |
| 23 | ??? |  | 10.17 | 1521 | 237.2 |  | no |
| 24 | ??? |  | 10.22 | 1527 | 184.1 |  | no |
| 25 | 1-methylcyclohexanecarboxylate ?? ^*)^ | aliphatic carboxylic acid | 10.26 | 1532 | 199.1 | 668 | no |
| 26 | ??? |  | 10.28 | 1534 | 122.0 |  | no |
| 27 | threonate | sugar acid | 10.37 | 1547 | 292.2 | 924 | yes |
| 28 | ??? |  | 10.47 | 1560 | 166.1 |  | no |
| 29 | ??? |  | 10.49 | 1563 | 157.0 |  | no |
| 30 | ??? |  | 10.52 | 1566 | 220.1 |  | no |
| 31 | proline^4)^ | amino acid | 10.64 | 1582 | 142.2 | 847 | yes |
| 32 | ??? |  | 10.67 | 1587 | 182.1 |  | no |
| 33 | ??? |  | 10.71 | 1592 | 217.1 |  | no |
| 34 | 2-amino-4,6-dihydroxypyrimidine ?? ^*)^ | heteroaromatic alcohol | 10.97 | 1626 | 328.1 | 684 | no |
| 35 | ??? |  | 10.99 | 1629 | 181.0 |  | no |
| 36 | ??? |  | 11.09 | 1642 | 157.2 |  | no |
| 37 | ??? |  | 11.13 | 1648 | 166.0 |  | no |
| 38 | ??? |  | 11.39 | 1683 | 228.0 |  | no |
| 39 | 2-methyl-4-tetradecene ?? | alkene | 11.48 | 1694 | 141.0 | 605 | no |
| 40 | ribonate ?? ^*)^ | sugar acid | 11.53 | 1701 | 129.0 | 686 | no |
| 41 | methyl tetradecanoate | fatty acid ester | 11.77 | 1733 | 143.2 | 907 | yes |
| 42 | ??? |  | 11.80 | 1736 | 123.1 |  | no |
| 43 | ??? |  | 11.85 | 1743 | 141.1 |  | no |
| 44 | ??? |  | 11.94 | 1756 | 138.2 |  | no |
| 45 | tagatose ?? | sugar | 11.99 | 1762 | 217.1 | 761 | no |
| 46 | ??? |  | 12.06 | 1771 | 223.1 |  | no |
| 47 | methyl galactoside ?? | sugar | 12.12 | 1780 | 204.2 | 610 | no |
| 48 | azelaate ?? | aliphatic dicarboxylic acid | 12.25 | 1797 | 204.0 | 658 | no |
| 49 | ??? |  | 12.26 | 1798 | 294.2 |  | no |
| 50 | ??? |  | 12.27 | 1799 | 101.0 |  | no |
| 51 | lyxose ?? ^*)^ | sugar | 12.34 | 1809 | 217.1 | 686 | no |
| 52 | ??? |  | 12.42 | 1819 | 294.2 |  | no |
| 53 | phytol ?? ^*)^ | diterpene alcohol | 12.47 | 1825 | 123.0 | 707 | no |
| 54 | ??? |  | 12.56 | 1837 | 157.2 |  | no |
| 55 | phytol^7)^ | diterpene alcohol | 12.59 | 1842 | 123.1 | 930 | yes |
| 56 | phytol^7)^ | diterpene alcohol | 12.76 | 1864 | 123.2 | 923 | yes |
| 57 | phytol^7)^ | diterpene alcohol | 12.90 | 1882 | 109.1 | 944 | yes |
| 58 | methyl hexadecatetraenoate ?? | fatty acid ester | 12.93 | 1886 | 105.1 | 788 | no |
| 59 | ??? |  | 13.14 | 1919 | 136.0 |  | no |
| 60 | methyl glucoside ?? ^*)^ | sugar | 13.16 | 1922 | 204.0 | 715 | no |
| 61 | methyl palmitate | fatty acid ester | 13.19 | 1929 | 143.1 | 920 | yes |
| 62 | ??? |  | 13.22 | 1933 | 136.1 |  | no |
| 63 | ethyl glucoside ?? | sugar | 13.25 | 1938 | 204.1 | 790 | no |
| 64 | ethyl palmitate | fatty acid ester | 13.64 | 2008 | 101.1 | 760 | yes |
| 65 | docosahexaenoate (C22:6) ? ^*)^ | fatty acid | 13.69 | 2016 | 105.1 | 842 | no |
| 66 | ??? |  | 13.99 | 2070 | 211.1 |  | no |
| 67 | docosahexaenoate (C22:6) ? ^*)^ | fatty acid | 14.07 | 2084 | 108.1 | 829 | no |
| 68 | methyl glucoside ? ^*)^ | sugar | 14.09 | 2087 | 204.0 | 819 | no |
| 69 | ??? |  | 14.19 | 2105 | 294.1 |  | no |
| 70 | methyl docosahexaenoate ? ^*)^ | fatty acid ester | 14.22 | 2110 | 105.1 | 800 | no |
| 71 | methyl stearidonate | fatty acid ester | 14.24 | 2115 | 105.0 | 924 | yes |
| 72 | methyl linolenate | fatty acid ester | 14.34 | 2132 | 108.1 | 865 | yes |
| 73 | xylose ? ^*)^ | sugar | 14.40 | 2143 | 204.1 | 819 | no |
| 74 | oleyl alcohol, trifluoroacetate ?? | fatty alcohol ester | 14.42 | 2146 | 124.0 | 762 | no |
| 75 | methyl glucoside ? ^*)^ | sugar | 14.50 | 2160 | 204.1 | 825 | no |
| 76 | ??? |  | 14.55 | 2170 | 197.1 |  | no |
| 77 | methyl eicosatetraenoate ?? | fatty acid ester | 14.64 | 2186 | 108.2 | 710 | no |
| 78 | phytol^7)^ | diterpene alcohol | 14.74 | 2202 | 143.1 | 882 | yes |
| 79 | eicosapentaenoate (C20:5) ? ^*)^ | fatty acid | 14.91 | 2233 | 105.1 | 835 | no |
| 80 | linolenate (C18:3) | fatty acid | 15.02 | 2253 | 107.2 | 902 | yes |
| 81 | 2-palmitoylglycerol ?? ^*)^ | fatty acid ester | 15.23 | 2289 | 239.2 | 663 | no |
| 82 | ethyl glucoside ?? | sugar | 15.28 | 2298 | 204.1 | 614 | no |
| 83 | ??? |  | 15.34 | 2309 | 246.0 |  | no |
| 84 | ??? |  | 15.38 | 2316 | 322.3 |  | no |
| 85 | ??? |  | 15.40 | 2320 | 204.2 |  | no |
| 86 | hexose glycoside | sugar derivative | 15.46 | 2330 | 204.0 | 855 | no |
| 87 | ??? |  | 15.56 | 2348 | 204.1 |  | no |
| 88 | eicosen-1-ol ?? | fatty alcohol | 15.60 | 2356 | 110.0 | 685 | no |
| 89 | 1-myristoylglycerol ?? | fatty acid ester | 15.98 | 2423 | 343.3 | 716 | no |
| 90 | ??? |  | 16.07 | 2437 | 354.3 |  | no |
| 91 | ??? |  | 16.12 | 2448 | 204.0 |  | no |
| 92 | ??? |  | 16.15 | 2452 | 313.1 |  | no |
| 93 | methyl docosahexaenoate | fatty acid ester | 16.38 | 2494 | 105.0 | 909 | yes |
| 94 | glucose ?? ^*)^ | sugar | 16.69 | 2548 | 204.2 | 605 | no |
| 95 | ethyl glucoside ? | sugar | 16.73 | 2555 | 204.1 | 828 | no |
| 96 | 1-palmitoylglycerol | fatty acid ester | 17.00 | 2603 | 371.3 | 805 | yes |
| 97 | cellobiose ?? | sugar | 17.31 | 2658 | 204.2 | 769 | no |
| 98 | trehalose | sugar | 17.75 | 2736 | 361.1 | 909 | yes |
| 99 | squalene | triterpene | 18.22 | 2816 | 121.2 | 898 | yes |
| 100 | lactose ?? ^*)^ | sugar | 18.32 | 2832 | 204.1 | 664 | no |
| 101 | 4-*O*-galactopyranosylmannopyranose ? ^*)^ | sugar | 18.92 | 2925 | 204.1 | 825 | no |
| 102 | lactose ?? ^*)^ | sugar | 19.28 | 2981 | 204.2 | 726 | no |
| 103 | cholestane-3,7,12,25-tetrol, tetraacetate ?? | sterol ester | 19.63 | 3035 | 253.1 | 665 | no |
| 104 | tocopherol (vitamin E) ?? | phenolic terpene | 20.24 | 3129 | 237.1 | 652 | no |
| 105 | turanose ?? ^*)^ | sugar | 20.97 | 3222 | 103.1 | 743 | no |
| 106 | ??? |  | 21.79 | 3289 | 204.1 |  | no |
| 107 | lycopene ?? | tetraterpene | 22.33 | 3333 | 351.2 | 639 | no |
| 108 | 3,5-cyclo-ergosta-7,9(11),22-triene-6-ol ?? | sterol | 22.44 | 3342 | 294.1 | 636 | no |
| 109 | 3,5-cyclo-ergosta-7,9(11),22-triene-6-ol ?? | sterol | 22.81 | 3372 | 143.1 | 668 | no |
| 110 | methyl glucoside ?? ^*)^ | sugar | 23.71 | 3446 | 204.1 | 747 | no |
| 111 | methyl glucoside ?? ^*)^ | sugar | 24.42 | 3503 | 204.1 | 731 | no |
