# Supplementary material for: Metabolic profiling identifies trehalose as an abundant and diurnally fluctuating metabolite in the microalga Ostreococcus tauri
Source: Metabolomics. 2017 Apr 17;13(6):68. doi: 10.1007/s11306-017-1203-1 (PMC5392535; doi:10.1007/s11306-017-1203-1)
Supplement: Supplementary file 7 — Supplementary material 7 (DOCX 11 KB) [file 11306_2017_1203_MOESM7_ESM.docx]

**Supplementary Table 4. List of gene model identifiers mentioned in this work.** For the listed proteins, information is provided for all predicted isoenzymes.

| Encoded protein | Gene identifier in v1 | Gene identifier in v2 |
| --- | --- | --- |
| Serine hydroxymethyltransferase | Ot03g00910 | ostta03g00920 |
| Serine hydroxymethyltransferase | Ot14g02820 | ostta14g02420 |
| Serine hydroxymethyltransferase | Ot19g00300 | ostta19g00170 |
| Squalene synthase | Ot04g02220 | ostta04g02080 |
| Bifunctional trehalose phosphate synthase-trehalose phosphatase (TPS-TPP) | Ot01g02410 | ostta01g02440 |
| Bifunctional trehalose phosphate synthase-trehalose phosphatase (TPS-TPP) | Ot12g02420 | ostta12g02400 |
| Trehalose phosphatase (TPP) | Ot14g00340 | ostta14g00250 |
| Ketopantoate hydroxymethyltransferase | Ot02g04650 | ostta02g02520 |
| Pantothenate synthetase | Ot09g02660 | ostta09g02630 |
| Acetyl-CoA carboxylase | Ot01g03240 | ostta01g03280 |
| Acetyl-CoA carboxylase | Ot10g03560 | ostta10g03450 |
